# Comparative cryopreservation of bovine and porcine primary hepatocytes

**DOI:** 10.3389/fvets.2023.1211135

**Published:** 2023-08-08

**Authors:** Sandra Andres, Babett Bartling, Vera Stiensmeier, Alexander Starke, Marion Schmicke

**Affiliations:** ^1^Institute of Agricultural and Nutritional Sciences, Animal Health Management, Martin Luther University Halle-Wittenberg, Halle (Saale), Germany; ^2^Department for Ruminants and Swine, Faculty of Veterinary Medicine, Leipzig University, Leipzig, Germany; ^3^Clinic for Cattle, Endocrinology, University of Veterinary Medicine Hannover, Hanover, Germany

**Keywords:** primary cells, liver, cattle, pig, controlled-rate freezer, freezing medium, trehalose

## Abstract

The isolation of primary hepatocytes from liver tissue of farm animals yields a very high number of cells, and a part of them can be stored by cryopreservation for future experiments. As no experience exists with the cryopreservation of hepatocytes from cattle, our study aimed at the cryopreservation of bovine hepatocytes by use of different protocols compared with the cryopreservation of hepatocytes from pig. We tested different freezing media (William’s Medium E vs. University of Wisconsin solution), cryoprotectants (dimethyl sulfoxide with vs. without trehalose as additional additive), freezing systems (standard freezing container vs. controlled-rate freezer) and freezing times (4 vs. 28 d). These tests identified a general influence of species and freezing systems, whereas the influence of freezing media, trehalose additive and freezing time was less or not obvious. In this regard, we determined a mean recovery of 30% of bovine hepatocytes and 55% of porcine hepatocytes cryopreserved in a controlled-rate freezer, whereas the rates were about 10% less when hepatocytes were frozen in a standard freezing container. In accordance with this observation, the cultivation of cryopreserved hepatocytes from cattle was less effective than that of porcine hepatocytes. Hepatocytes from cattle can be successfully cryopreserved and partially cultured after cryopreservation but with lower percentage than porcine hepatocytes.

## Introduction

Hepatocytes are liver parenchymal cells and mainly contribute to total liver mass. They are highly functional cells being responsible for various anabolic and catabolic pathways, and the detoxification of exogenous and endogenous substances. Cell cultures of primary hepatocytes derived from human and rodent are important *in vitro* models to study basic mechanisms in these cells (1, 2). Moreover, they are utilized in medical, pharmaceutical and toxicological applications thereby replacing or, at least, reducing *in vivo* studies (1, 2). Basic and application-related research is also performed by use of hepatocytes isolated from farm animals, especially pig and chicken (2–4). As hepatocytes derived from cattle are seldomly used in research studies, our group established a model of primary bovine hepatocytes in monoculture and in coculture with other hepatic cells to reduce this gap (5–7).

Hepatocytes were commonly isolated from liver tissue by enzymatic digestion of the tissue with a crude collagenase fraction containing collagenolytic and proteolytic activities (2). In case of liver tissue from cattle and pig, this yields in a very high number of hepatocytes due to the relatively big mass of the liver. As not all of them can be used in one experimental setting, many hepatocytes remain and could be stored for later experiments.

A common method of storing cells including hepatocytes for a longer time is the cryopreservation by freezing cells in cryoprotective agents below −130°C (8–10). Cryoprotective agents prevent the formation of intracellular ice crystals and reduce the osmotic stress caused by cellular dehydration during the freezing process (10, 11). They are classified into penetrating and nonpenetrating cryoprotective agents. Penetrating agents [e.g., glycerine, dimethyl sulfoxide (DMSO), ethylene glycol, propylene glycol] enter through the cell membrane and prevent the formation of intracellular ice crystals, whereas nonpenetrating agents (e.g., glucose, sucrose, trehalose, polyvinylpyrrolidone) remain extracellularly and reduce the osmotic stress (10, 11). Both penetrating and nonpenetrating agents also reduce cellular damages during the thawing process.

The most efficient cryoprotective agent for human and animal cells is the organic solvent DMSO and, therefore, mostly used for freezing cells including primary hepatocytes (10, 11). In contrast to penetrating cryoprotectants, nonpenetrating cryoprotectants are less applied in routine. However, several studies indicate the beneficial effect of particularly trehalose in reducing the osmotic stress during freezing and thawing cells (12–14).

Cryoprotectants are always diluted in freezing medium with 5–20% final concentration in case of DMSO (10, 11). Freezing media usually correspond to the cell type-specific cell culture media. They are often supplemented with fetal bovine serum (FBS) (10, 11). Moreover, the University of Wisconsin solution is frequently used as freezing medium (15, 16). This solution was developed in the 1980s for clinical application (16). As it allows a proper preservation of human liver grafts, the University of Wisconsin solution often serves as freezing medium for the cryopreservation of hepatocytes (8, 15).

Finally, cells suspended in freezing medium with cryoprotectant become frozen at −80°C in a styrofoam or alcohol-filled freezing container both allowing a slow and permanent freezing rate (17). The freezing rate in such containers is still too fast in the temperature range above −10°C and, therefore, intracellular ice crystals are formed whereas it is too slow in the temperature range below −10°C and, therefore, cells undergo high osmotic stress (17–19). As also heat of solidification is released when a substance changes from liquid to solid, the temperature in the container does not correspond to that in the cryovial (17, 18, 20). Freezing cells in such containers is therefore a rather uncontrolled technique. These problems have been solved by the introduction of controlled-rate freezers to cryopreserve cells (17). Controlled-rate freezers deliver liquid nitrogen into a closed chamber into which the cryovial with cell suspension is placed, and the cell type-specific freezing rate is programmed and well monitored by use of a reference vial (17).

The controlled-rate freezer system is also used for the cryopreservation of hepatocytes freshly isolated from human and pig (18–22). However, there is no experience with the cryopreservation of hepatocytes from cattle. Hepatocytes of ruminants like cattle differ in their cellular metabolism from hepatocytes of nonruminant animals (23). Therefore, our study aimed at the cryopreservation of hepatocytes from cattle by use of different freezing media, cryoprotectants and freezing systems, and its comparison to the cryopreservation of hepatocytes from pig.

## Materials and methods

### Animals

This study used healthy liver tissues from donor animals that were euthanized because of either unfavorable prognosis or experimental reason (file numbers 33.9-42502-04-18/2752 and H1-2//1-20 of the local animal welfare commissions). Donor animals were Holstein Friesian cattle (3 weeks to 7 years of age) and domestic pigs (8–10 weeks of age) of both sexes. Euthanasia was performed with pentobarbital at two clinical departments (Department for Ruminants and Swine, Faculty of Veterinary Medicine, Leipzig University; Clinic for Cattle, University of Veterinary Medicine Hannover) and at the Research Centre for Agricultural and Nutritional Sciences of the Martin Luther University Halle-Wittenberg. Immediately after determination of cardiorespiratory arrest and subsequent surgical opening of the abdominal cavity, liver tissue of the caudate lobe (30 to 40 g) was dissected and well rinsed with ice-cold Hank’s Balanced Salt Solution (HBSS) supplemented with HEPES, NaHCO_3_, CaCl_2_, MgCl_2_, MgSO_4_, nutrients, and heparin (500 I.E./l). The rinsed tissue was then kept in this HBSS/HEPES/NaHCO_3_-based buffer on ice until hepatocyte isolation in the local laboratory unit.

### Hepatocyte isolation and culture

**Figure 1:**
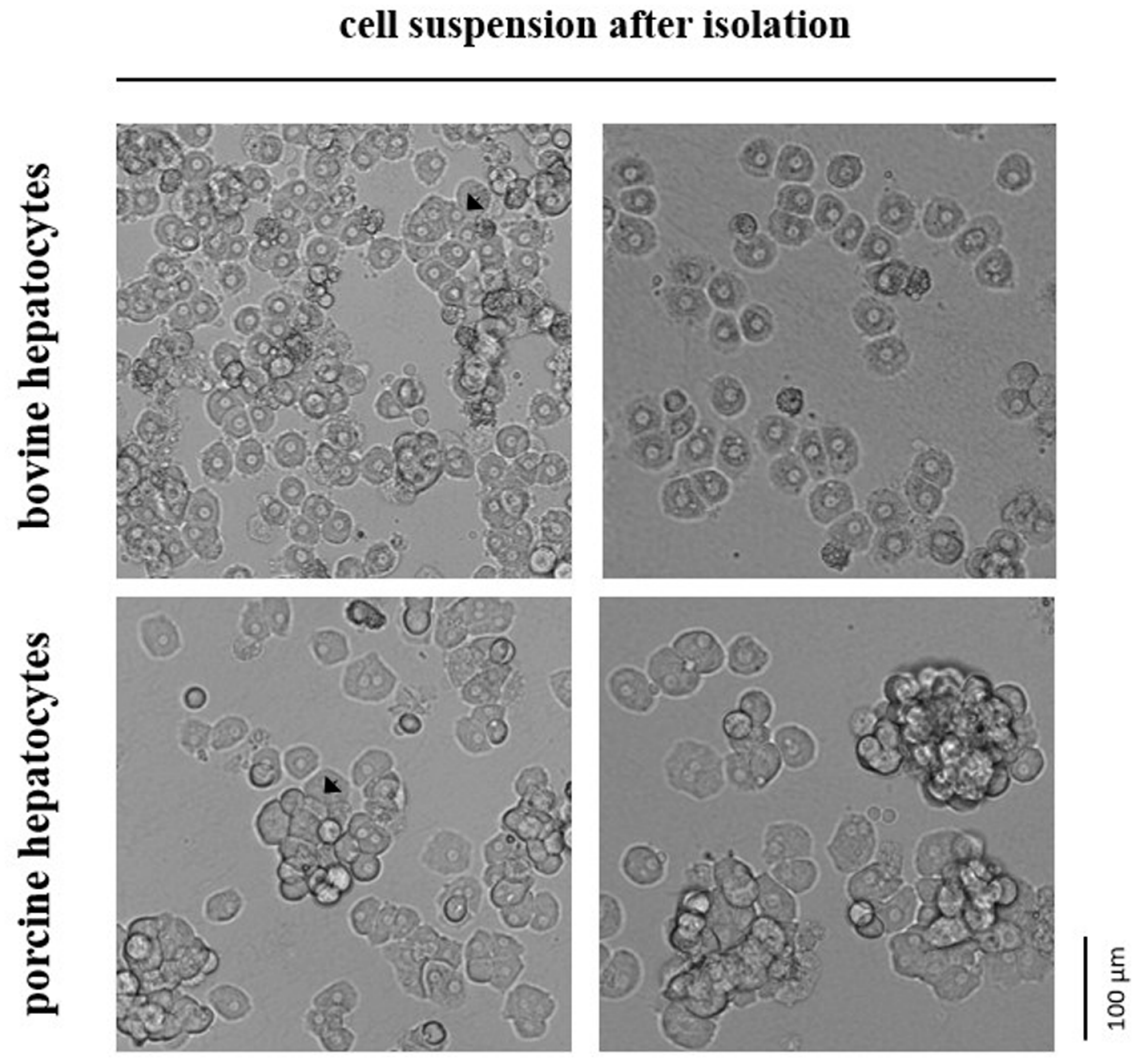
In suspension hepatocytes immediately after enzymatic isolation from the liver tissue of cattle or pig. Arrows indicate selected spheroids formed by the primary hepatocytes.

Primary hepatocytes from cattle and pig were isolated 2.0–4.0 h and 1.5–3.5 h, respectively, after liver dissection according to an established protocol (6) with some modifications. In brief, the cold liver tissue was rewarmed to 37°C by rinsing it with warm HBSS/HEPES/NaHCO_3_ buffer and then subjected to a two-step-collagenase perfusion. The perfusion was performed with HBSS/HEPES/NaHCO_3_ buffer supplemented with EGTA for 5 min, CaCl_2_ for 5 min and finally CaCl_2_ and collagenase (1.5 Wünsch units/g liver tissue) for 5–7 min until the tissue was soft. Collagenase NB 4G (Nordmark Pharma GmbH, Uetersen, Germany) was used for digesting bovine liver, whereas Collagenase P (Roche, Mannheim, Germany) was used for porcine liver. Collagenase NB 4G had less damaging effect on the hepatocytes from cattle than collagenase P, but it was not digestive enough for porcine liver tissue (preliminary observations). Moreover, we used less EGTA for perfusing the bovine liver (0.5 mM) than the porcine liver (1.0 mM) because of size differences in the hepatocyte aggregations after isolation (Figure 1). After perfusion, hepatocytes were extracted by cutting the liver tissue with a scalpel and swiveling it in William’s Medium E (PAN-Biotech, Aidenbach, Germany) that was supplemented with 20% FBS (PAN-Biotech). Finally, hepatocytes were filtered through a 200-μm nylon mesh, centrifuged at 60 × g for 3 min and resuspended in William’s Medium E with 10% FBS. All steps were performed at 37°C.

The number and vitality of hepatocytes was assessed by staining the cells with 0.4% trypan blue solution (Sigma-Aldrich, Deisenhofen, Germany) and counting trypan blue-positive and -negative cells separately in a Neubauer cell counting chamber. While most hepatocytes were then subjected to cryopreservation procedure, a small part of the hepatocytes was freshly seeded on 12-well plates (140 × 10^3^ vital cells/cm^2^) that had been coated with rat tail tendon collagen (Roche, Mannheim, Germany). Hepatocytes were cultured in William’s Medium E with 10% FBS, 1 × insulin-transferrin-selenium solution (Sigma-Aldrich) and 40 ng/mL dexamethasone (Sigma-Aldrich) for first 4 h and afterwards in the same medium without FBS.

### Hepatocyte cryopreservation

We used two different basic media for cryopreservation: William’s Medium E and University of Wisconsin solution (Bridge to Life Ltd., London, United Kingdom). Each basic medium was supplemented with 40% FBS, 20% DMSO (PanReac Applichem, Darmstadt, Germany) and, in selected cases, 200 mM trehalose (Carl Roth, Karlsruhe, Germany) to prepare a 2 × cryopreservation solution. The DMSO concentration of 20% in the 2 × cryopreservation solution has been identified as optimal in preliminary experiments (not shown). As the hepatocytes are suspended in William’s Medium E with 10% FCS after cell isolation, they were centrifuged at 60 × *g* for 1 min and then resuspended in a respective volume of either ice-cold William’s Medium E or ice-cold University of Wisconsin solution to reach a concentration of 2 × 10^6^ vital cells/ml. Thereafter, this cell suspension was stepwise mixed on ice with the same volume of the respective 2 × cryopreservation solution to reach a 1 × solution, aliquoted (1 × 10^6^ vital cells/cryovial) and transferred to the cryopreservation process. The cryopreservation process was performed either in a propan-2-ol-filled freezing container (Nalgene^®^ Mr. Frosty; Thermo Fisher Scientific, Waltham, MA, United States) placed at −80°C or in a controlled-rate freezer (Consarctic GmbH, Westerngrund, Germany) cooling down to −100°C. Propan-2-ol-filled freezing containers placed at −80°C reach a cooling rate of about −1°C/min, whereas the controlled-rate freezer starts with a lower cooling rate followed by shock cooling step and finishes with a higher cooling rate (Supplementary Figure). Our protocol used for control-rate freezing corresponds to that reported for rat hepatocytes (20) and resembles to that reported for porcine hepatocytes (18). All hepatocytes were subsequently transferred to and stored in vapor phase liquid nitrogen (below −135°C).

Hepatocytes from cattle or pig were thawed 4 and 28 d after cryopreservation. In detail, each cryovial was swiveled in hot water (80°C) until 80% of the cell suspension was thaw. The cell suspension was then transferred into a larger vial and stepwise diluted with ice-cold William’s Medium E containing 10% FBS within 3 min. Thereafter, the hepatocytes were centrifuged (30 × *g* for 3 min), resuspended in ice-cold William’s Medium E with 10% FBS and subjected to the cell counting procedure. Moreover, hepatocytes were seeded and cultured on 12-well plates coated with collagen as described above.

### Statistics

All hepatocyte cryopreservations were performed in duplicate. The relative number of vital cells of a total cell fraction was calculated as number of trypan blue-negative cells per number of trypan blue-negative and -positive cells × 100%. The recovery rate of hepatocytes was calculated as number of trypan blue-negative cells before cryopreservation per number of trypan blue-negative cells after cryopreservation × 100%. Statistics and data presentation were performed by use of the software OriginPro 2019 (OriginLab Corporation; Northampton, MA). Significant differences (*p* ≤ 0.05) between two or more groups were tested by ANOVA with Bonferroni *post-hoc* test. For detail, see figure legends.

## Results

Our study assessed the total number and cell viability of bovine and porcine hepatocytes by cell staining with trypan blue solution followed by manual microscopic cell counting. The manual counting technique was preferred over automated techniques because hepatocytes often remain in stable cell aggregations (i.e., spheroids) after isolation. These spheroids were commonly smaller and less frequently formed in case of hepatocytes isolated from cattle compared with hepatocytes from pig (Figure 1). However, the proportion of living and dead hepatocytes directly after isolation from liver tissue did not differ between both species (mean of all tests performed; Table 1). After cryopreservation and subsequent thawing process the relative number of hepatocytes with intact and, therefore, trypan blue-impermeable cell membrane decreased with higher decrease for bovine than porcine hepatocytes (Table 1).

**Table 1:** Trypan blue-assessed cell viability before cryopreservation and after thawing of primary hepatocytes from cattle and pig.

Species	Trypan blue-negative hepatocytes per total cell fraction (%)		
	Before	After	Difference
Cattle	87 ± 7	50 ± 17^*^	−36 ± 19
Pig	87 ± 3	63 ± 13^*,#^	−24 ± 13^#^

Data are means ± SD (*n* = 96 using 6 animals for each species) with *p* < 0.001 vs. ^*^before of the same species and ^#^cattle of the same test group (two-way ANOVA with Bonferroni *post-hoc* test).

**Figure 2:**
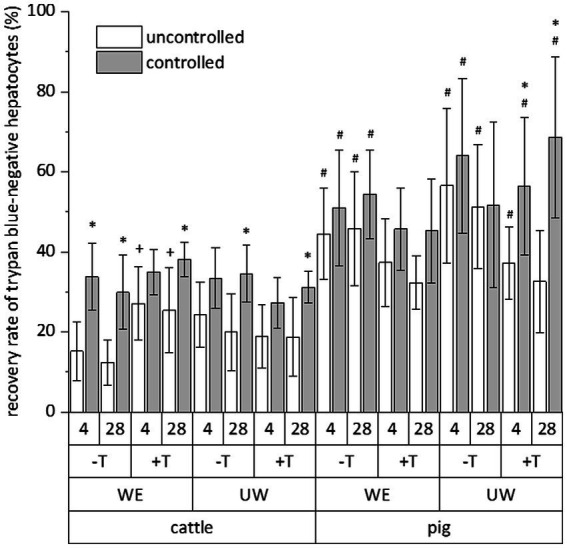
Comparative influence of cryopreservation by uncontrolled (propan-2-ol-filled freezing container) or controlled (controlled-rate freezer) techniques on the recovery rate of primary hepatocytes from cattle or pig, which have been frozen with (+) or without (−) addition of trehalose (T) in DMSO/FCS-containing William’s medium E (WE) or University of Wisconsin solution (UW) and stored below −135°C for 4 or 28 days. This evaluation only included hepatocyte with intact cell membrane after staining with trypan blue solution. Data are means ± SD (*n* = 6) with *p* < 0.001 for species and freezing process (multiple three-way ANOVA tests) and *p* < 0.05 vs. ^*^uncontrolled freezing process, ^#^cattle, and ^+^without trehalose of the individual test group (Bonferroni *post-hoc* test).

**Figure 3:**
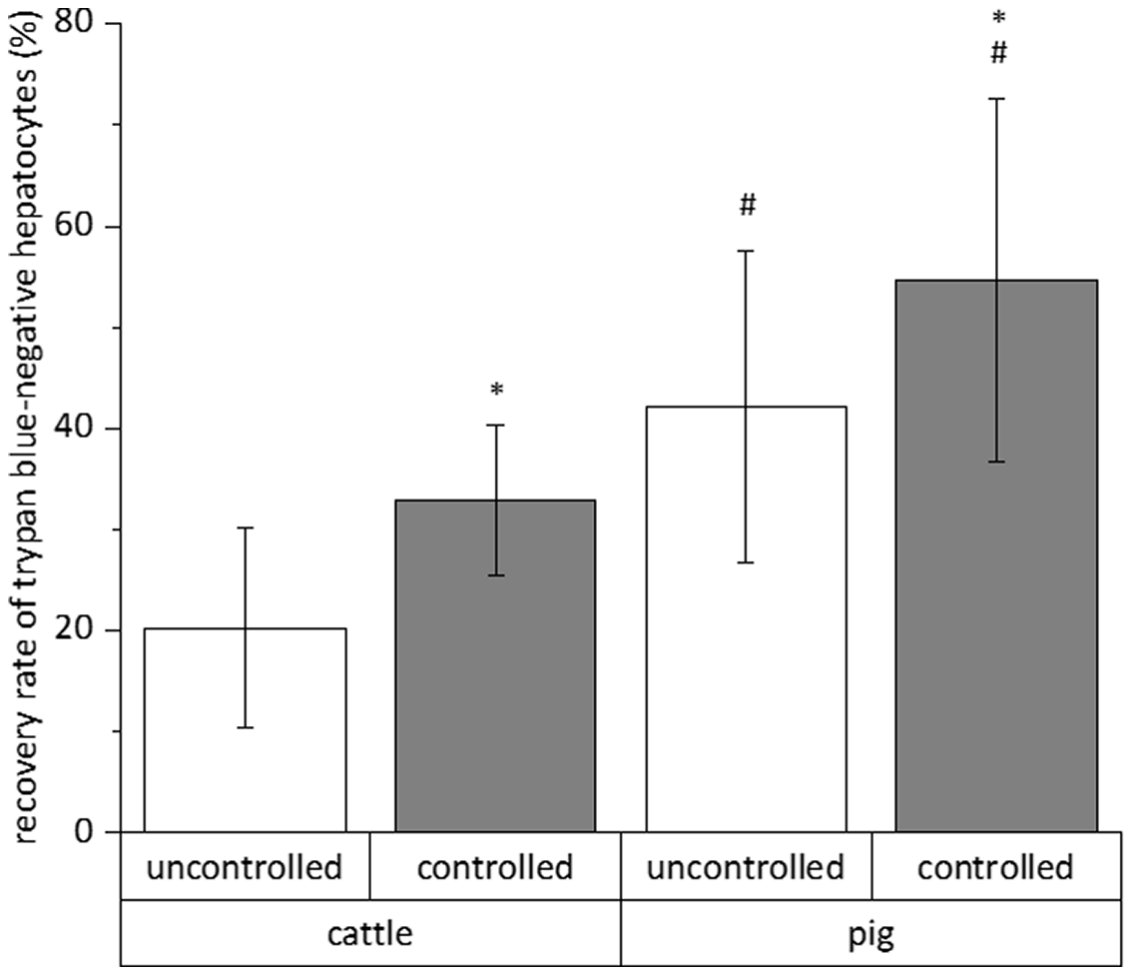
Mean influences of freezing process and species on the recovery rate of primary hepatocytes after cryopreservation in different solutions for 4 and 28 days. We only counted hepatocytes with intact cell membrane after cell staining with trypan blue solution. Data are means ± SD (*n* = 48 using 6 animals each species) with *p* < 0.001 vs. ^*^uncontrolled and ^#^cattle (two-way ANOVA with Bonferroni *post-hoc* test).

The relative number of vital cells of a total cell fraction is no absolute parameter indicating the success of a cryopreservation procedure. Therefore, we calculated the recovery rate of exclusively trypan blue-negative cells to assess the simultaneous influence of species (cattle and pig), freezing system (uncontrolled and controlled), freezing medium (William’s E medium and University of Wisconsin solution each supplemented with DMSO and FCS), trehalose supplementation and time (4 and 28 days) on the cryopreservation of hepatocytes. All data of the individual cryopreservation tests are summarized in Figure 2. This summary indicates a sole effect of species or freezing system on the recovery rate of trypan blue-negative hepatocytes, whereas time or freezing medium have no significant effect (Figure 2). The post-cryopreservation recovery of bovine hepatocytes was commonly lower than the recovery of porcine hepatocytes (Figure 2). Nevertheless, hepatocytes from cattle often responded better to the beneficial effect of the controlled freezing system than hepatocytes from pig (Figure 2). In only one case, the supplementation of the freezing medium with trehalose had an additional benefit on the recovery rate (see hepatocytes from cattle in William’s E medium; Figure 2). Because of the primary effect of species or freezing system on the recovery of primary hepatocytes after cryopreservation, Figure 3 shows the recovery of trypan blue-negative cells in dependency of these both parameters only.

**Figure 4:**
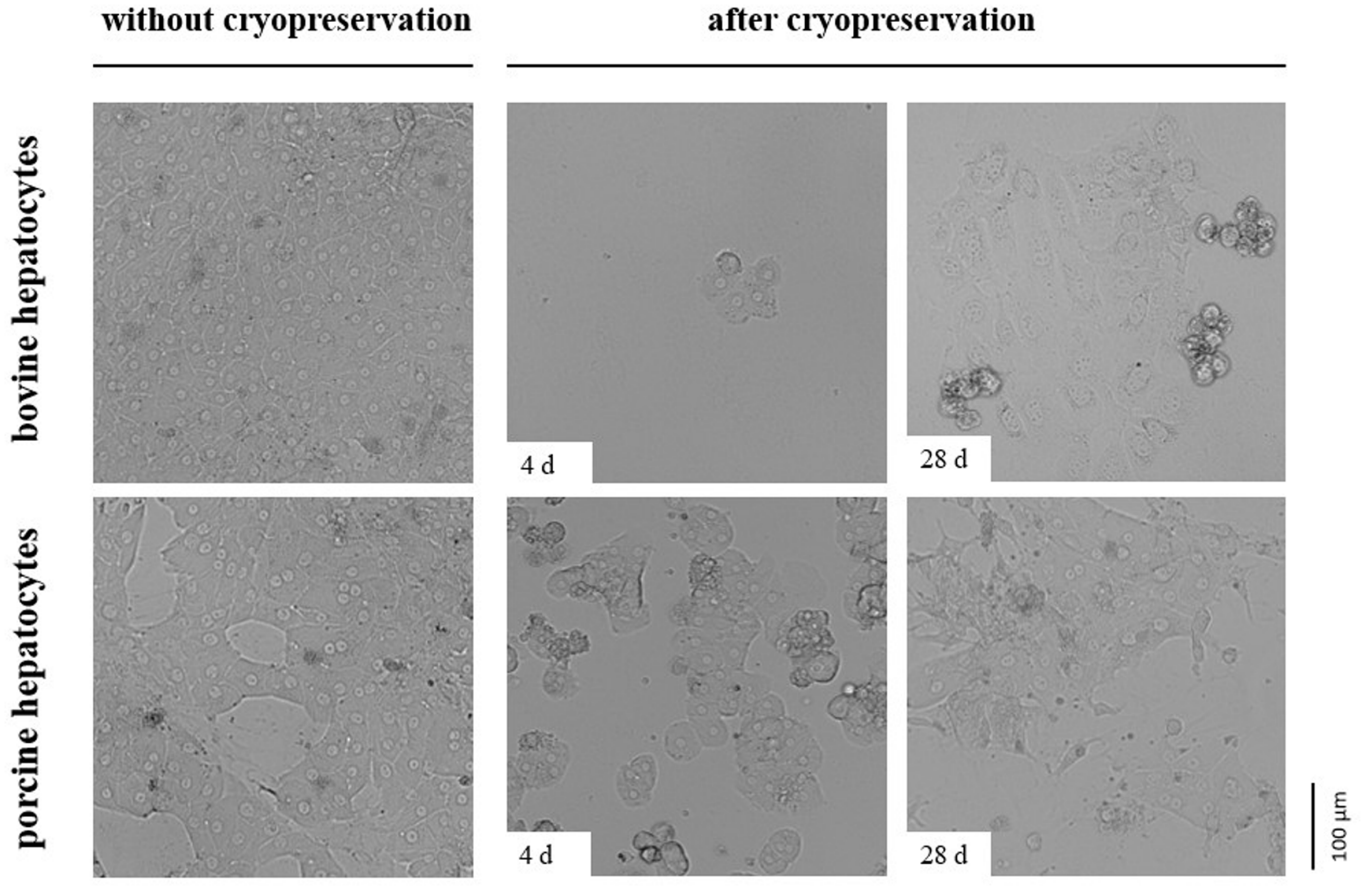
Comparative images showing primary hepatocytes from cattle or pig seeded on a collagen layer directly after cell isolation (without cryopreservation) or after controlled cryopreservation in DMSO/FCS-containing University of Wisconsin solution with trehalose. Cryopreservation was performed for 4 or 28 d. All cells were imaged 2 days after seeding.

The impermeability of cells to trypan blue or other life/dead stains at the time of investigation does not indicate cell viability and functionality later in culture. As hepatocytes are well polarized cells adhering with their basolateral cell membrane to extracellular matrix compounds and adjacent cells, we additionally tested the post-cryopreservation capability of our hepatocytes to cell adherence and spreading. These tests revealed that the number of bovine hepatocytes showing proper cell adherence and spreading on collagen after cryopreservation is less than the number of porcine hepatocytes (Figure 4). However, the number of porcine hepatocytes that can be successfully cultured after cryopreservation was too little to reach confluent cell layers (Figure 4).

## Discussion

This study showed that hepatocytes freshly isolated from cattle can be successfully cryopreserved and partially cultured after cryopreservation. However, the relative number of bovine hepatocytes recovering from the cryopreservation is low and much lower compared with cryopreserved hepatocytes from pig. Although the recovery rate was commonly better when hepatocytes from both animals were frozen in a controlled-rate freezer than in an alcohol-filled freezing container, this rate was not high enough to reach confluent cell layers for subsequent cell cultivations. The supplementation of the freezing medium with trehalose improved the cryopreservation of bovine hepatocytes only if William’s medium E served as basic medium, whereas trehalose had no beneficial effect if supplemented to the University of Wisconsin solution. Porcine hepatocytes did not profit from the supplementation with trehalose. Moreover, we did not determine clear differences between both basic media William’s medium E and University of Wisconsin solution as well as the time of cryopreservation.

The enzymatic digestion of the liver tissue results in the release of single hepatocytes and hepatocyte spheroids of different size. However, this release is not only mediated by the enzymatic action of the crude collagenase fraction used for hepatocyte isolation but also by the preceding perfusion of the liver tissue with EGTA. EGTA is a chelating agent with high affinity for Ca^2+^ ions. Its use irreversibly disrupts Ca^2+^-dependent desmosomes, specialized cell surface structures for cell-to-cell adhesion (24). In 2012, Lee and colleagues clearly showed that porcine hepatocytes aggregated in spheroids recover better from the cryopreservation than single hepatocytes (22). As hepatocytes isolated from cattle form smaller or even no spheroids after isolation compared with porcine hepatocytes, it eventually explains their lower post-cryopreservation recovery rate. This species-dependent difference in the spheroid size could not be equalized by use of a lower EGTA concentration and another crude collagenase fraction having lower proteolytic activity during the isolation procedure of bovine hepatocytes. This observation suggests significant differences in cell–cell adhesion complexes between hepatocytes of cattle and pig. Eventually, porcine hepatocytes express more Ca^2+^-independent desmosomes or more other cell–cell junctions (i.e., adherence junctions, thigh junctions) than bovine hepatocytes. Also, species-dependent differences in cell-matrix junctions are well possible.

Another species-dependent difference is the hepatic metabolism of cattle and pig (23). However, we used the same basic media (i.e., William’s medium E and University of Wisconsin solution) for freezing bovine and porcine hepatocytes. As both media had been developed for conserving or culturing primary hepatocytes of non-ruminant species, their use could additionally explain the lower post-cryopreservation recovery of bovine hepatocytes. Although this suggests the development of cattle-specific basic media for freezing hepatocytes, the basic medium is eventually not that critical because cell storages below −135°C minimize the hepatic metabolism to a very low degree. This assumption is indirectly supported by several studies showing no differences in the post-cryopreservation recovery of human or rat hepatocytes frozen at highly divergent concentrations of FBS (0 to 90%) and, therefore, divergent proportions of the basic medium (15, 25, 26).

The recovery rate of the hepatocytes after cryopreservation highly depends on their viability at the time of isolation from the liver tissue. Our study assessed the cell viability by trypan blue staining of the hepatocytes. Although this common method did not reveal differences between the viability of bovine and porcine hepatocytes immediately after isolation from the liver tissue, trypan blue staining only assesses the integrity of the outer plasma membrane of cells. Therefore, species-dependent differences in cell death pathways preceding outer plasma membrane damages could also be responsible for the less successful cryopreservation of bovine hepatocytes compared with porcine hepatocytes. Mean differences in animals age and time between liver dissection and hepatocyte isolation are possible reasons for species-related differences in cell death mechanisms upstream of membrane damages. However, the cryopreservation of bovine hepatocytes has been partially improved by supplementation of the freezing medium with trehalose thereby confirming its potential benefit as non-penetrating cryoprotective agent (12–14).

In addition to the species-dependent differences in the post-cryopreservation recovery of hepatocytes from cattle and pig, we commonly observed better recovery rates if the hepatocytes were frozen under controlled conditions in a controlled-rate freezer. This observation supports the advantage of this technology in the cryopreservation of hepatocytes (18–22). About 50–60% of the porcine hepatocytes frozen under controlled conditions recovered from the cryopreservation. This rate corresponds to findings of other studies using pigs (15, 18, 22). However, the recovery rate further decreases when hepatocytes were transferred to cell culture systems. This observation has been made by us and other researchers using hepatocytes of various species (15, 18, 27–31). The low temperature-mediated downregulation of β1-integrin is one crucial factor explaining the limited cell cultivation of cryopreserved hepatocytes because it essentially contributes to cell-matrix interactions (27). Another crucial factor is the cytotoxicity of DMSO (11).

In summary, our study showed that the cryopreservation of bovine hepatocytes is possible but less successful than the cryopreservation of porcine hepatocytes. Moreover, we demonstrated a limited cultivation of cryopreserved hepatocytes of both species compared to rat hepatocytes. Therefore, novel cryoprotectants and cryopreservation protocols adapted to these novel agents are required for the cryopreservation of primary hepatocytes from cattle and pig as well. Regarding the recovery of cryopreserved hepatocytes in post-thaw culture, additional assays such as staining thawed cells for apoptotic markers may further elucidate the cause of poor cell recovery in post-thaw culture.

## Data availability statement

The original contributions presented in the study are included in the article/Supplementary material, further inquiries can be directed to the corresponding author.

## Ethics statement

The animal study was reviewed and approved by the use of animal material was accepted by the local animal welfare commissions under file numbers 33.9-42502-04-18/2752 and H1-2//1-20.

## Author contributions

MS, SA, and BB contributed to conception and design of the study. SA organized the database and wrote sections of the manuscript. SA and VS performed the experiments. BB performed the statistical analysis and wrote the first draft of the manuscript. All authors contributed to manuscript revision, read, and approved the submitted version.

## Funding

This work was supported by the European Union’s Horizon 2020 project “BovReg” under grant number 815668.

## Conflict of interest

The authors declare that the research was conducted in the absence of any commercial or financial relationships that could be construed as a potential conflict of interest.

## Publisher’s note

All claims expressed in this article are solely those of the authors and do not necessarily represent those of their affiliated organizations, or those of the publisher, the editors and the reviewers. Any product that may be evaluated in this article, or claim that may be made by its manufacturer, is not guaranteed or endorsed by the publisher.
