# Supplementary material for: Comparative cryopreservation of bovine and porcine primary hepatocytes
Source: Front Vet Sci. 2023 Aug 8;10:1211135. doi: 10.3389/fvets.2023.1211135 (PMC10442649; doi:10.3389/fvets.2023.1211135)
Supplement: Supplementary file 1 [file Image_1.pdf]

Supplementary Material

Comparative cryopreservation of bovine and porcine primary hepatocytes

Sandra Andres, Babett Bartling, Vera Stiensmeier, Alexander Starke, Marion Schmicke*

*** Correspondence:** Corresponding Author: Marion.Schmicke@tiho-hannover.de

1 Supplementary Figures

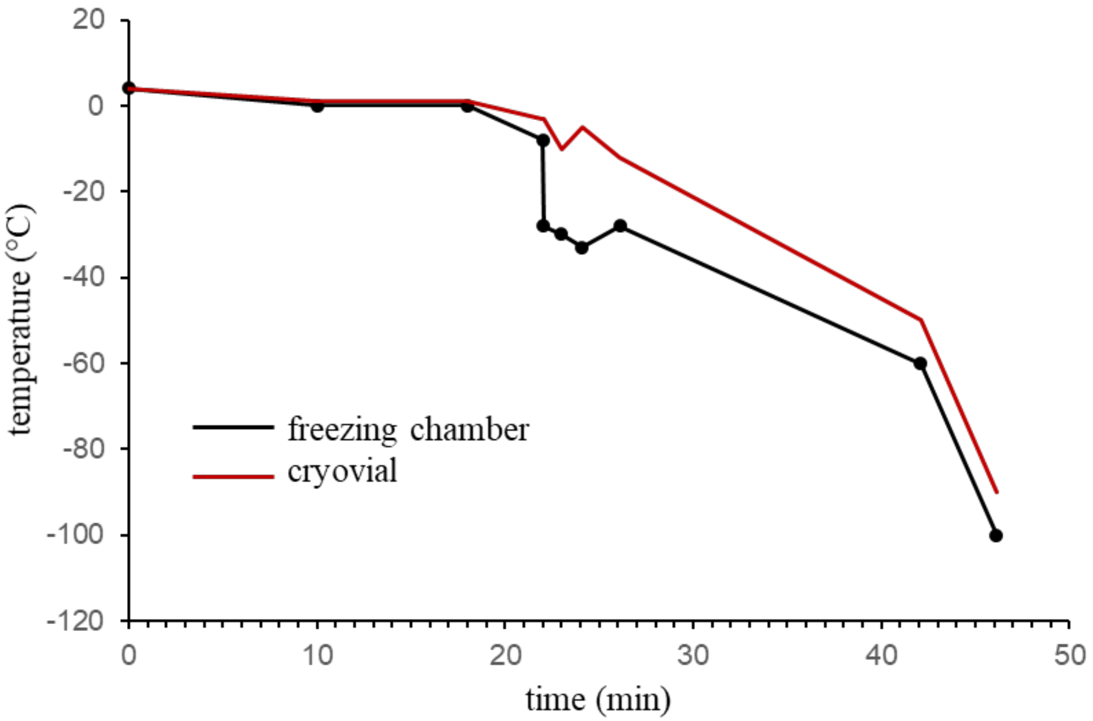

Supplementary Figure. Comparative temperature profile in the freezing chamber of the controlled-rate freezer and hepatocyte-containing cryovial during the control-rate freezing process.
